# The Role of Psychological Capital and Intragroup Conflict on Employees' Burnout and Quality of Service: A Multilevel Approach

**DOI:** 10.3389/fpsyg.2016.01755

**Published:** 2016-11-14

**Authors:** Jose M. Leon-Perez, Mirko Antino, Jose M. Leon-Rubio

**Affiliations:** ^1^Universidad de Sevilla, Social PsychologySevilla, Spain; ^2^Instituto Universitário de Lisboa (ISCTE-IUL)Lisbon, Portugal

**Keywords:** psychological capital, workplace conflict, conflict management, well-being, performance

## Abstract

Previous studies have found a negative association between intragroup conflict and both employees' health and performance, including the quality of service that employees provide. However, some authors have indicated that such negative effects of intragroup conflict depend on how conflict is managed. In addition, at individual level, research is increasingly emphasizing the role of psychological strengths (i.e., psychological capital) as predictors of health and performance. Thus, this research addresses both a main effect at individual level (psychological capital on burnout/quality of service) and a moderated cross-level model (2-2-1: intragroup conflict, conflict management climate and burnout/quality of service) in a cross-sectional survey study (*N* = 798 workers nested in 55 units/facilities). Results revealed a main effect of psychological capital on both burnout (*r* = −0.50) and quality of service (*r* = 0.28). Also, there was an association between intragroup relationship conflict and burnout (*r* = 0.33). Finally, there was an interaction effect in which conflict management climate buffers the negative association between intragroup conflict and quality of service. Practical implications of these results for developing positive and healthy organizations that prevent potential psychosocial risks at group level while promote individual strengths are discussed.

## Introduction

Traditionally, the absence of distress at work together with the development of health promotion initiatives has been considered pivotal indicators of healthy organizations. However, recent developments in the field of psychology have led to a shift in the ultimate goal of occupational health research: from reducing negative events at work and their concomitant psychological, physical, and economic costs to facilitate positive experiences that promote health and well-being (e.g., Bono et al., 2013). Indeed, recent trends in organizational behavior focus on the concept of positive and healthy organizations (PHO), which refers to such organizations that emphasize the importance of achieving positive organizational outcomes (i.e., financial success, high quality service, and organizational survival) by promoting employees' flourishing in a healthy work environment over time (e.g., Cooper and Cartwright, 1994; Grawitch et al., 2006; Day and Randell, 2014).

PHO has its roots in the Human Relations Movement, which (a) proposes that satisfied workers are more productive than others (i.e., “the happy-productive worker hypothesis”: Wright et al., 2007), and (b) calls for a psychology focusing on building organizations that facilitate employee development and promote quality of life (i.e., positive psychology: Seligman and Csikszentmihalyi, 2000). Thus, research on “this area focuses on building strengths at work rather than fixing weaknesses” both at the individual and organizational level (for a review, see Gilbert and Kelloway, 2014).

However, Hackman (2009) cautioned that this positive approach, when applied to organizational context, has too much emphasis on individual-level interventions and ignores problems in organizations that need attention prior to introducing positive interventions. Furthermore, Bono et al. (2013) pointed out the lack of integration of both the traditional approach focusing on preventing resource-depleting (negative) experiences and their associated negative outcomes (e.g., distress and health complaints), and the more recent positive approach focusing on facilitating resource-building (positive) experiences and their associated positive outcomes (e.g., work engagement and thriving).

In order to shed some light on this debate (see also Fineman, 2006; Roberts, 2006), this study uses a cross-level design to address how both psychological strengths at individual level (i.e., PsyCap) and both negative and positive experiences at group level (i.e., intragroup conflict and conflict management climate) interplay and are associated to employees' well-being (i.e., reduced burnout) and performance (i.e., self-reported quality of service). In doing so, we first focus on psychological capital (i.e., positive psychological resources) as a relevant factor contributing to workers' well-being and performance at individual level, particularly we assume that workers' psychological capital is an essential resource at individual level both to cope with job demands or work stressors and to trigger positive experiences at work, which are key features of positive and healthy organizations. Then, considering the pivotal role of teams in nowadays organizations, we turn to a group level of analysis and emphasize how positive group dynamics, such as the capacity of the team to openly discuss disagreements and constructively manage conflict, can both prevent negative environments and foster positive social interactions in order to enhance employees' well-being and performance.

### PHO at individual level

There is a renewed interest in the value of positively oriented human resource strengths and psychological capacities that allow individuals and organizations to flourish (e.g., Avey et al., 2008; Bakker and Schaufeli, 2008). According to this positive approach, Luthans et al. (2007) and (Luthans et al. (2015) have proposed the concept of psychological capital (PsyCap), which is a multidimensional construct consisting of hope (positive subjective appraisals of goal-related capabilities), efficacy (confidence in one's own abilities to successfully perform tasks), resiliency (positive coping and bouncing back from adversity), and optimism (mental attitude to interpret situations and events in a positive way).

From a stress prevention perspective, Folkman and Moskowitz (2000) indicated that positive emotions have an adaptive function under stress conditions because they “may provide a psychological break or respite, support continued coping efforts, and replenish resources that have been depleted by the stress” (p. 649: see also Fredrickson, 2001). In a similar vein, resilience and optimism, components of psychological capital, appear to play an important role in the capacity to tolerate and copy with stressful events, and have been associated with reduced stress (e.g., Ong et al., 2006; Carver and Scheier, 2014). In that sense, Baron et al. (2016) found that PsyCap increases well-being through the reduction of perceived stress in a sample of entrepreneurs. Furthermore, a recent meta-analysis summarizing data from 51 independent samples, documented a significant negative relationship between PsyCap and both job stress and anxiety (Avey et al., 2011).

Similarly, some studies have confirmed the negative association between PsyCap and burnout in various professions such as school teachers (Cheung et al., 2011), bank employees (Li et al., 2015), or nurses (Ding et al., 2015); which suggests that having these psychological resources can help workers tolerating stressful situations or confront their demands at work without suffering chronic stress or burnout. Moreover, Laschinger and Fida (2014) conducted a two-wave survey with Canadian newly graduated nurses and found that nurses' PsyCap was related to both lower initial levels of burnout and lower increases in burnout after their first year of practice.

In addition, these psychological resources have been widely reported in the literature as precursors of well-being and happiness (e.g., Lyubomirsky et al., 2005; Wood et al., 2011). In organizational contexts, according to Hobfoll's (1989, 2001, 2002) Conservation of Resource (COR) theory, several authors have considered PsyCap as cumulative resource gains that increase well-being over time (gain spirals: Avey et al., 2010; Culbertson et al., 2010). More recently, longitudinal studies have associated PsyCap with increased overall well-being and work happiness over time (e.g., Luthans et al., 2013; Williams et al., 2015) and a meta-analysis has shown that PsyCap is a significant predictor of psychological well-being and other desirable employee attitudes (e.g., job satisfaction: Avey et al., 2011).

Moreover, the role of PsyCap can go beyond reducing stress at work to increasing the positive experience of work and improving work engagement and performance. For example, Peterson et al. (2011) conducted a longitudinal study with three data collection times from a large financial service organization. Their results revealed that PsyCap is related to both objective (i.e., sales revenue) and subjective performance (i.e., supervisor ratings) over time. Also, Avey et al. (2011) meta-analysis reported a significant positive relationship between PsyCap and multiple measures of performance (self, supervisor evaluations, and objective). Furthermore, the effect size of such relationship was stronger in the service sector compared to those organizations based in the manufacturing industry, probably because performance in the service sector “relies more on social interactions that require emotional norms favoring the expression of positive affect” (Avey et al., 2011, p. 146). However, although quality of service can be considered a competitive advantage related to organizations' productivity as it enhances customer satisfaction and build a long-term relationship with customers (e.g., Rust et al., 2002), to authors' knowledge no previous study has explored the impact of PsyCap on the quality of service that workers provide to their customers.

Nevertheless, based on previous literature on the association between PsyCap and performance, it seems that people with higher PsyCap will succeed in providing a high quality of service because they will perceive they have necessary skills to perform their tasks (self-efficacy) and being successful (optimism). Hence, these cognitive schemas will allow them to put the necessary effort to (self-efficacy), redirect their courses of action to (hope), and being persistent to (resilience) successfully providing high-quality of service. For example, empirical evidence indicates that positive cognitive judgments about ones' capabilities (e.g., self-efficacy beliefs) and cognitive abilities (e.g., emotional intelligence) are crucial regulatory mechanisms for effective performance in a wide range of tasks that require specific social competencies (for a meta-analysis see Stajkovic and Luthans, 1998; Joseph and Newman, 2010; although for contradictory results see also the meta-analysis of Sitzmann and Yeo, 2013).

Building on these rationales and findings, we propose that PsyCap is negatively associated to burnout (H1) and positively associated to perceived quality of service (H2).

### PHO at group level

Following the idea that PHO are organizations that both prevent or successfully manage negative environments and group dynamics and foster positive social interactions in order to enhance employees' well-being and performance, we turned our focus to the role of workplace conflict, which is inherent to social interactions at work and constitutes one of the most important sources of stress at workplace. Indeed, Keenan and Newton (1985) using an open-ended method found that interpersonal conflict was one of the most frequently reported sources of stress in a sample of young engineers. Also at the individual level, Spector and Jex (1998) conducted a meta-analysis and concluded that interpersonal conflict at work is related to individual negative consequences such as frustration, anxiety, and depression. Similarly, more recent cross-sectional and diary studies have shown that interpersonal conflict can be considered a job stressor that leads to high levels of stress and correlates with stress-related outcomes (i.e., strain) such as anxiety, emotional exhaustion or psychosomatic complaints (e.g., Dijkstra et al., 2009, 2011; Meier et al., 2014).

However, these studies are at individual level while an interpersonal conflict is usually a group process (i.e., intragroup conflict) that affects the whole team or unit. Indeed, autonomous working groups for delivering high-quality products and services are on the most common ways of designing work in current organizations because collaboration usually yields superior outcomes compared to individual efforts (Deutsch and Coleman, 2000). In addition, these studies have rarely taken into consideration the typology of intragroup conflict based on their nature (Jehn, 1997; Jehn and Mannix, 2001): (a) *task-related conflict* (TC) or conflicts concerning the perception of disagreement among individuals about the content of their inter-related tasks, which usually involves differences in points of view, ideas and opinions; (b) *relationship conflict* (RC) or conflicts concerning perceptions of interpersonal incompatibilities, usually including gossip and disagreement about personal beliefs; and (c) *process conflict* (PC) or conflicts concerning controversies about how task accomplishment will proceed, usually involving disagreement about procedures, protocols and guidelines.

According to such typology and considering conflict at group level, recent meta-analyses have highlighted that such types of intragroup conflict have different consequences (see De Dreu and Weingart, 2003; de Wit et al., 2012): while relationship and process conflict have negative relationships with personal and group outcomes (i.e., well-being and performance), task conflict can be productive under certain circumstances depending on how the group conceive conflict and deal with it (i.e., conflict management climate or employees' shared beliefs that disagreement can be discussed and intragroup conflicts are generally managed well and fairly in their unit: Einarsen et al., 2016). For example, relationship and process intragroup conflict can be detrimental to the quality of service that employees provide because it deteriorates the group's affective climate (Gamero et al., 2008) and service climate (Benítez et al., 2012).

On the other hand, task conflict can improve performance when teams have open discussion norms (Jehn and Mannix, 2001) and a climate of psychological safety exists (Bradley et al., 2012). Consequently, several authors have highlighted that the way how conflict is managed determines its outcomes rather than the kind of conflict (e.g., Behfar et al., 2008; Tekleab et al., 2009). Similarly, Tjosvold (2008) argued that teams need to rely on cooperative management of conflict for their successfully internal functioning, which can have significant benefits for both individuals and organizations, including increased performance as recent follow-up training studies have suggested. For example, employees working in a call center who were trained for cooperative conflict management improved their performance (i.e., fewer turnover rates, more calls answered, fewer customer complaints, and better quality examination scores) after the training compared to their non-trained colleagues (Tjosvold et al., 2014). Also, Leon-Perez et al. (2016) trained 258 health-care workers in cooperative conflict management skills and reported that the training was successful in reducing the number of patients' complaints and the level of absenteeism of trained workers, whereas their non-trained colleagues working in the same Spanish hospital exhibited no corresponding changes over time. At team level, teams working in a Chinese leading high-technology company reported higher creativity and productivity after being trained for cooperative teamwork and constructive controversy (Lu et al., 2010).

These results are in line with previous research showing that teams discussing their differences openly and constructively can improve decision-making processes and relational bonds, helping teams to resolve their conflicts satisfactorily and therefore being more innovative (Chen et al., 2005; Song et al., 2006), increasing their performance (Alper et al., 2000; Behfar et al., 2008; Tekleab et al., 2009; Somech et al., 2009), and preventing conflict escalation or potential negative cycles of hostilities among members (Greer et al., 2008; Leon-Perez et al., 2015). Moreover, a recent meta-analysis concluded that “Conflict processes, that is, *how* teams interact regarding their differences, are at least as important as conflict states, that is, the *source and intensity* of their perceived incompatibilities” (DeChurch et al., 2013, p. 559).

Thus, we hypothesize a cross-level main effect in which intragroup conflict is positively associated to burnout (H3: relationship conflict H3a; process conflict H3b; task conflict H3c) and negatively associated to perceived quality of service (H4: relationship conflict H4a; process conflict H4b; whereas task intragroup conflict has a positive relationship with quality of service H4c). Additionally, we propose that the conflict resolution mechanism of the group (i.e., conflict management climate) buffers the positive association between intragroup conflict and burnout (H5: relationship conflict H5a; process conflict H5b; task conflict H5c) as well as the negative association between intragroup conflict and quality of service (H6: relationship conflict H6a and process conflict H6b; whereas conflict management climate enhances the positive association between task intragroup conflict and quality of service: H6c).

**Figure 1:**
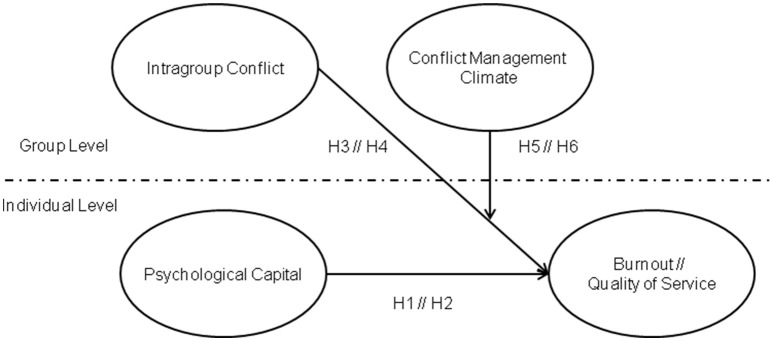
**Research model**.

In sum, this study seeks to contribute to the existing literature by examining the interplay between individuals' strengths and group dynamics in fostering employees' well-being and performance. In doing so, we try to overcome limitations in previous research that has tended to treat working conditions and group processes as relatively stable characteristics of an environment, often neglecting their dynamic and multilevel nature. Our hypotheses are graphically represented in Figure 1. The confirmation of these hypotheses may have relevant practical implications for developing positive and healthy organizations that prevent potential psychosocial risks at group level while promoting individual strengths.

## Methods

### Sample and procedure

After obtaining the consent of the company's CEO, data was gathered in a vehicle safety and emissions inspection company in Spain. Participation in this cross-sectional survey study was voluntary and confidential. According to the American Psychological Association's (APA) Ethical Principles of Psychologists and Code of Conduct, participants were informed about the aim of the study and requisites for participation, and all participants gave their written informed consent. Indeed, surveys were administered to groups of workers in company time with a research assistant present to answer any questions. Participants placed their completed questionnaires in a sealed box to ensure the anonymity of responses. From the 815 returned questionnaires (response rate = 61.7%) we obtained 798 valid questionnaires. Thus, hypotheses were tested in a sample of 798 workers nested in 55 units/facilities (*M* = 35.87 years old; *SD* = 8.49; 81.8% male; 94.5% permanent full-time contracts), who reported job tenure of 9.77 years (*SD* = 6.37).

### Measures

#### Psychological capital (PsyCap)

We used the Psychological Capital Questionnaire (Luthans et al., 2007) in its 12-items reduced version validated in Spanish (Leon-Perez et al., in press). This questionnaire measures a composite higher-order construct termed as *psychological capital (PsyCap)*, consisting of four positive psychological states: efficacy (confidence in one's own abilities to successfully perform tasks), hope (positive subjective appraisals of goal-related capabilities), resiliency (positive coping and bouncing back from adversity), and optimism (mental attitude to interpret situations and events in a positive way). The 12 items of this questionnaire (e.g., “I feel confident analyzing a long-term problem to find a solution”; “If I should find myself in a jam at work, I could think of many ways to get out of it”) were presented with a 6-point Likert-type scale (1 = completely disagree, 6 = completely agree). Reliability estimated through Cronbach's Alpha was satisfactory (α = 0.85).

#### Intragroup conflict

Intragroup conflict was measured using the Intragroup Conflict Scale in its 14-item version (Jehn et al., 2008) translated into Spanish following a back-translation procedure. This scale includes three types of intragroup conflicts: *task conflict* or disagreements related to the content of the tasks that workers have to perform (e.g., “How much did this team have to work through disagreements about varying opinions?”), *relationship conflict* or disagreements about personal issues [e.g., “We disagreed about non-work (social or personality things,”)] and *process conflict* or disagreements regarding the planning and delegation issues of task accomplishment (e.g., “How much disagreement was there about task responsibilities within this team?”). All items were rated on a 5-point Likert-type scale ranging from 1 = “almost never” to 5 = “very often.” Reliability estimated through Cronbach's Alphas were satisfactory for all the three dimensions of the intragroup conflict: 0.89 for task, 0.85 for relationship, and 0.92 for process, respectively.

#### Conflict management climate

The existing measures of conflict management at team level lack of validation into Spanish and usually refer either to the conflict management styles the team uses to solve conflicts (e.g., cooperative vs. competitive: Somech et al., 2009; reactive vs. preemptive: Tekleab et al., 2009) or directly to the effective resolution of conflicts (e.g., conflict resolution: Greer et al., 2008) but not to both essential components for effective conflict management in teams: cooperation and effective resolution (Tjosvold, 2008). In response, we used a single item that captures employees' shared beliefs that disagreement can be discussed and intragroup conflicts are generally managed well and fairly in their unit (i.e., “In my team/unit conflict arises as in all teams everywhere; however, my team openly discusses disagreements and conflicts are effectively managed and solved”).

#### Burnout

Burnout was measured with the Shirom-Melamed Burnout Measure (SMBM: Shirom and Melamed, 2006) in its Spanish version available from Shirom's personal website (http://www.shirom.org/arie/index.html). This measure consists of 14 items that are measured on a 7-point Likert scale (ranging from 1 = *never* to 7 = *always*) that comprise the physical (6 items: e.g., “I feel tired”), cognitive (5 items: e.g., “I have difficulty concentrating”), and emotional (3 items: e.g., “I feel I am not capable of investing emotionally in coworkers and customers”) dimensions of being burned-out. Reliability estimated through Cronbach's Alpha was satisfactory (α = 0.95).

#### Quality of service

Quality of Service was measured with a self-reported 10-item questionnaire developed by Salanova et al. (2005) to measure perceived quality of service through three dimensions: service climate (e.g., “Employees in our organization have knowledge of the job and the skills to deliver superior quality work and service,”) empathy (e.g., “Employees understand specific needs of customers”) and excellent job performance (e.g., “Employees deliver an excellent service quality that is difficult to find in other similar organizations.”) All items were scored on a 7-point rating scale ranging from 1 (completely agree) to 5 (completely disagree). Reliability estimated through Cronbach's Alpha was satisfactory (α = 0.89).

#### Control variables

Beyond socio-demographic variables such as age, sex, and job tenure, we included the Positive and Negative Affect Schedule (PANAS: Watson et al., 1988) in its Spanish version (Sandín et al., 1999) to measure participants' affect. It comprises two mood scales, one measuring positive affect and the other measuring negative affect. Each item is rated on a 5-point scale ranging from 1 = *very slightly or not at all* to 5 = *extremely* to indicate the extent to which the respondent usually feels this way. In this study, Cronbach's Alphas were satisfactory for both dimensions: 0.74 for positive affect, and 0.70 for negative affect, respectively. This variable was used as control since previous studies have shown its association to conflict experiences (see Griffith et al., 2014).

## Results

### Descriptives and correlations among variables

First, we computed the internal consistency of each measure as well as Means, standard deviations and correlations among the main variables in this study, as shown in Table 1 for the variables at the first level of analysis (worker) and in Table 2 for the variables aggregated at the second level (units or facilities); in this second table we also include the aggregation indexes.

**Table 1:** **Descriptive statistics and bivariate correlations among the level 1 variables of the study (***N*** = 798)**.

**Variables**	***M***	***SD***	**Age**	**Sex**	**Con**.	**PA**	**NA**	**Psy**	**QoS**	**BN**
Age	35.87	8.49	−							
Sex	0.82	0.39	−0.02	−						
Contract	0.95	0.23	−0.14^**^	−0.13^**^	−					
Pos. Aff.	3.01	0.50	−0.21^**^	0.04	0.13^**^	(0.74)				
Neg. Aff.	2.17	0.49	−0.10^**^	0.09^*^	0.04	0.32^**^	(0.70)			
PsyCap	4.83	0.59	−0.10^**^	−0.05	0.11^**^	0.42^**^	−0.12^**^	(0.85)		
Qual. Serv	3.57	0.68	−0.06	−0.07	0.14^**^	0.31.^**^	−0.11^**^	0.43^**^	(0.89)	
Burnout	2.31	1.16	0.06	−0.01	0.01	−0.34^**^	0.32^**^	−0.45^**^	−0.46^**^	(0.95)

*reliability estimated with Cronbach's alpha is presented in the diagonal between parentheses*.

* *p < 0.05*,

** *p < 0.01*.

**Table 2:** **Descriptive statistics, aggregation indexes and bivariate correlations among the level 2 variables of the study (55 units/facilities)**.

**Variables**	***M***	***SD***	***ICC1***	***ICC2***	**TC**	**RC**	**PC**	**CMC**
Task conflict	2.15	0.32	0.132	0.620	−			
Relationship conflict	1.40	0.28	0.155	0.662	0.55^**^	−		
Process conflict	1.87	0.40	0.170	0.686	0.82^**^	0.50^**^	−	
Conflict management climate	2.85	0.31	0.152	0.543	−0.33^**^	−0.29^**^	−0.34^**^	−

** *p < 0.01*.

### Tests of the hypotheses

To test our hypotheses 1 and 3 that included burnout as an outcome or dependent variable, we employed hierarchical linear modeling (HLM), using the software SPSS. We tested a first model (Model 1) entering our control variables, namely sex, role within the company, age, as well as positive and negative affects. In Model 2 we added our first level predictor psychological capital. In Model 3 we included our second level (units or facilities) predictors, which were task conflict, relationship conflict and process conflict. This procedure is based on recent recommendations in the literature (Aguinis et al., 2013).

Table 3 reports the parameters of interest of the different models used to test Hypothesis 1 and 3. Before estimating the 3 mentioned models, we checked for the amount of variance of Burnout that is attributable to the between units/facilities differences: 13.2%, a proportion worthy to be studied with a multilevel approach (Hayes, 2006).

**Table 3:** **HLM results to test main and cross-level effects on burnout (H1, H3)**.

	**Model 1 Estimation (SE)**	**Model 2 Estimation (SE)**	**Model 3 Estimation (SE)**
Intercept	3.08^**^(0.38)	5.13(0.46)	2.45(0.36)
**WORKER LEVEL**			
Sex	0.05(0.18)	0.05(0.16)	−0.00(0.13)
Age	0.00(0.01)	0.00(0.01)	0.00(0.01)
Role	−0.03(0.13)	−0.01(0.13)	−0.09(0.09)
Positive affects	−1.08^**^(0.09)	−0.80^**^(0.09)	−0.81^**^(0.06)
Negative affects	1.09^**^(0.09)	0.93^**^(0.09)	0.91^**^(0.09)
Psychological capital		−0.50^**^(0.07)	−0.50^**^(0.07)
**UNIT LEVEL**			
Relationship conflict			0.33^†^(0.20)
Process conflict			−0.00(0.36)
Task conflict			0.14(0.25)
**ADDITIONAL INFORMATION OF THE MODEL ESTIMATION**			
−2 log likelihood (FIML)	1640.77^**^	1591.16^**^	1589.27^**^
Number of estimated parameters	9	10	13

*N = 798 operators nested in 55 groups/facilities. Standard errors in parentheses*.

† p < 0.10;

** *p < 0.01*.

Among the control variables, we did not find significant effects for sex, age and role within the organization. Nevertheless, positive affect was negatively related to burnout (*r* = −1.08, *p* < 0.01) while negative affect was positively related to burnout (*r* = 1.09, *p* < 0.01). In support of hypothesis 1, which stated a negative relationship between psychological capital and burnout, we found a significant and negative relationship (*r* = −0.50; *p* < 0.01). Regarding hypothesis H3a, which stated a positive effect of relationship conflict on burnout, we found a weak but worthy to be mentioned relationship (*r* = 0.33, *p* < 0.10); however, we did not found a significant relationship either between process conflict and burnout (H3b, *r* = −0.01, *p* = ns) nor between task conflict and burnout (H3c, *r* = 0.14, *p* = ns).

To test hypothesis H5, we allowed the slope to vary for each type of conflict and we introduced the Conflict Management Climate first as a predictor (Step 1) and then (Step 2) its interaction with the different type of conflicts (relationship conflict for H5a, process conflict for H5b, and task conflict for H5c). However, we did not find any empirical support for H5a, H5b and H5c. For reasons of space, results have not been reported but are available under request.

A similar data analysis strategy was used for testing hypotheses 2, 4, and 6, which included quality of service as dependent variable or outcome. First, before estimating the 3 mentioned models in an HLM analysis using SPSS, we checked for the amount of variance of quality of service that is attributable to the between units/facilities differences and found a similar value as for burnout: 11.6%.

As shown in Table 4, among the control variables, we did not find significant effects for sex, age, and role within the organization. Nevertheless, positive affect was positively related to quality of service (*r* = 0.50, *p* < 0.01) while negative affect was negatively related (*r* = −0.37, *p* < 0.01). In support of hypothesis H2, which stated a positive relationship between psychological capital and quality of service, we found a significant and positive relationship (*r* = 0.28; *p* < 0.01). Regarding hypothesis H4a, which stated a negative effect of relationship conflict on the quality of service, we found a weak but worthy to be mentioned relationship (*r* = −0.21, *p* < 0.10). Similarly, hypothesis H4b was supported as we found a significant negative relationship between process conflict and quality of service (*r* = −0.29, *p* < 0.05). However, regarding hypothesis H4c, which stated a positive effect of task conflict on the quality of service, we did not found a significant relationship (*r* = 0.09, *p* = ns).

**Table 4:** **HLM results to test main and cross-level effects on quality of service (H2, H4)**.

	**MODELS**		
	**Model 1 Estimation (SE)**	**Model 2 Estimation (SE)**	**Model 3 Estimation (SE)**
Intercept	2.52^**^(0.12)	1.82(0.31)	2.45(0.36)
**WORKER LEVEL**			
Sex	−0.07(0.14)	−0.07(0.11)	−0.03(0.11)
Age (centered)	0.00(0.01)	0.00(0.01)	0.00(0.01)
Role	−0.09(0.09)	−0.10(0.09)	−0.09(0.09)
Positive affects	0.50^**^(0.05)	0.35^**^(0.06)	0.35^**^(0.06)
Negative affects	−0.37^**^(0.05)	−0.30^**^(0.04)	−0.27^**^(0.06)
Psychological capital		0.28^**^(0.04)	0.27^**^(0.05)
**UNIT LEVEL**			
Relationship conflict			−0.21^†^(0.12)
Process conflict			−0.29^*^(0.12)
Task conflict			0.09(0.16)
**ADDITIONAL INFORMATION OF THE MODEL ESTIMATION**			
−2 log likelihood (FIML)	1188.57^**^	1176.07^**^	1148.52^**^
Number of estimated parameters	9	10	13

*N = 798 operators nested in 55 units/facilities. Standard errors in parentheses*.

† p < 0.10;

* p < 0.05;

** *p < 0.01*.

To test hypothesis H6, we allowed the slope to vary for each type of conflict and we introduced the Conflict Management Climate first as a predictor (Step 1) and then (Step 2) its interaction with the different type of conflicts (relationship conflict for H6a, process conflict for H6b, and task conflict for H6c). As shown in Table 5, regarding H6a the interaction was close to be significant (*r* = 0.07, *p* < 0.10) and its inclusion in the model (Step 2) reduced the negative impact of relationship conflict on quality of service (Step 1: *r* = −0.20, *p* < 0.10; Step 2: *r* = −0.16, *p* = ns). Given that both the effect of relationship conflict on quality of service as well as the interaction were significant only at *p* < 0.10, we can only affirm that we found a weak moderating effect and consequently a poor empirical support for H6a, but still worthy to be described as a tendency. Similarly, regarding H6b, the interaction was significant (*r* = 0.09, *p* < 0.05) and reduced the negative impact of process conflict on quality of service (Step 1: *r* = −0.27, *p* < 0.05; Step 2: *r* = −0.18, *p* = ns), so we can conclude that we found empirical support for H6b. Finally, regarding H6c, there was a non-significant main or interaction effect (Step 1: *r* = 0.10, *p* = ns; Step 2: *r* = 0.16, *p* = ns), concluding that we found no empirical support for H6c.

**Table 5:** **The moderating effect of Conflict Management Climate on the relationship between relationship, process, and task conflict on quality of service**.

	**Hypothesis tested**		
	**H6a Step 2**	**H6b Step 2**	**H6c Step 2**
Intercept	2.32(0.46)	2.45(0.36)	1.89^**^(0.48)
**INDIVIDUAL LEVEL**			
Sex	0.01(0.11)	−0.00(0.13)	−0.02(0.11)
Age	0.00(0.01)	0.00(0.01)	0.00(0.01)
Role	−0.09(0.09)	−0.09(0.09)	−0.09(0.09)
Positive affects	0.35^**^(0.06)	−0.81^**^(0.06)	0.34^**^(0.06)
Negative affects	−0.26^**^(0.06)	0.91^**^(0.09)	−0.26^**^(0.06)
Psychological capital	0.26^**^(05)	−0.50^**^(0.07)	0.26^**^(05)
**GROUP LEVEL**			
Task conflict (TC)	0.10(0.14)	0.14(0.25)	0.10(0.15)
Relationship conflict (RC)	−0.16(0.12)	0.22^†^(0.20)	−0.20^†^(0.12)
Process conflict (PC)	−0.13^**^(0.12)	−0.00(0.36)	−0.27^*^(0.12)
Conf. management climate (CMC)	0.14(0.09)	0.16(.10)	0.21^†^(0.09)
Interaction CMC^*^ RC	07^†^(0.10)		
Interaction CMC^*^ PC		09^*^ (.08)	
Interaction CMC^*^ TC			16^*^(0.28)
**ADDITIONAL INFORMATION OF THE MODEL ESTIMATION**			
−2 log likelihood (FIML)	1151.26^**^	1148.64^**^	1291.77^**^
Number of estimated parameters	14	14	12

*N = 798 operators nested in 55 units/facilities. Standard errors in parentheses*.

† p < 0.10;

* p < 0.05;

** *p < 0.01*.

In sum, results supported the proposed main effects of psychological capital (PsyCap) on burnout (H1) and quality of service (H2) as well as cross-level main effects of intragroup conflict on burnout (H3, only for relationship conflict: H3a) and quality of service (H4, for both relationship conflict and process conflict: H4a and H4b). Additionally, our results revealed that the conflict management climate of the unit did not moderate the relationship between intragroup conflict and burnout (H5), whereas the units' conflict management climate buffers the negative association between both relationship (H6a) and process conflict (H6b) and quality of service.

## Discussion and conclusion

This study tries to integrate the traditional approach focusing on preventing resource-depleting (negative) experiences (i.e., intragroup conflict) and the more recent positive approach focusing on facilitating resource-building (positive) experiences (i.e., conflict management climate, psychological capital) in order to improve employees' well-being and performance (i.e., decreased burnout and increased quality of service), which are considered key outcomes for developing positive and healthy organizations (PHO). In doing so, a multilevel approach is taken into consideration because represents better the integration between traditional approaches focusing on working conditions and group processes (unit level) and the positive psychology framework which emphasizes personal strengths and resources (individual level).

In that sense, our results revealed that PsyCap is a relevant cognitive component that has great potential for explaining key workplace outcomes such as reduced burnout and increased quality of service. This is a particularly encouraging result given the fact that PsyCap can be trained (e.g., Luthans et al., 2010; Dello Russo and Stoykova, 2015), and has therefore the potential to further overcome limitations associated to other potential interventions focusing on cognitive resources such as self-regulation mechanisms, which are considered a limited resource and a weak predictor of performance (for a meta-analysis see Sitzmann and Yeo, 2013). Thus, developing employees PsyCap may lead them to a positive psychological state of development that is not only positively related to well-being and health-related outcomes (e.g., lower levels of cholesterol: Luthans et al., 2013) but can also prevent distress at work (i.e., reduced burnout). According to the stress appraisal theory (Lazarus and Folkman, 1984), the stressor-strain relationship is determined by individuals' evaluation of both the situation (primary appraisal: the significance of what is happening for their well-being) and their perception of having available coping resources to manage such situation (secondary appraisal: coping options). Drawing on such theory, PsyCap can help employees to perceive their working demands as challenging instead of potentially harmful (primary appraisal guided by their optimism and hope) as well as their self-efficacy and resilience may lead them to consider that they can effectively cope with such demands at work, experiencing more positive than negative emotions such as anxiety or fear and therefore reducing psychological distress.

In addition, PsyCap is associated to improved quality of service. This result can be related to the previous one: PsyCap is associated to reduced burnout. In that sense, in line with “the happy-productive worker hypothesis” (Wright et al., 2007), Taris and Schreurs (2009) argued that burnout impedes performance because “high levels of emotional fatigue result in being unable as well as being unwilling to perform well” (p. 123). Thus, PsyCap can enhance performance because allow employees to experience positive emotions and reduced distress. Complementarily, Fredrickson (2001) suggests that positive emotions function to “broaden and build” skills and social capital, likely improving job performance and quality of service in social contexts such as work designs based on collaborative teams. Thus, employees with high PsyCap may deliver a higher quality of service because they experience more positive emotions, are more motivated and may expend more effort to perform well, which is in line with previous findings on the relationship between PsyCap and job performance at individual level (Avey et al., 2011).

Regarding the role of intragroup conflict, results revealed that only conflict about personal issues or relationship conflict is positively associated to burnout. This is in line with previous findings on conflict literature about the negative effects of relationship conflicts within the workplace. For example, Meier et al. (2013), in a daily diary study involving more than 4300 observations from 131 participants, found that daily relationship conflict predicted anger and well-being until the next day when task conflict was low, whereas task conflict was not related to anger and well-being. Similarly, from a conflict escalation perspective, several authors have found an association between relationship conflict and negative emotional reactions that lead to increased behaviors of mutual hostility that are part of conflict escalation (e.g., De Dreu and Van Vianen, 2001; Benítez et al., 2012; Arenas et al., 2015). Indeed, there is consensus about the negative effects of relationship conflict on employees' health and well-being since it usually implies more negative emotions such as anxiety, irritability, frustration, or tension compare to process and task-related conflict (for a meta-analysis see De Dreu and Weingart, 2003; de Wit et al., 2012). This can also explain why results did not reveal a moderation effect of conflict management climate on the association between relationship conflict and burnout: when conflict is about personal issues is more complex to manage and de-escalate to more productive levels (e.g., Arenas et al., 2015), resulting in impaired well-being. In contrast, neither task nor process conflict at team level was associated with burnout at individual level. Consequently, the existing conflict management climate did not show any moderation effect. A plausible explanation for these results is that such types of conflicts are usually associated with lower levels of tension and anxiety (i.e., are less stressful) compared to relationship conflicts, and therefore have non-significant associations with burnout, which can be considered as a long-term stress response.

On the other hand, as expected, results highlight that although relationship and process conflict can be detrimental to the quality of service that teams provide (de Wit et al., 2012), conflict management climate buffers the negative impact of both relationship and process conflict on such quality of service. Considering that positive psychology applied to organizational contexts “seek to identify the role of organizational climate and human resource practices (e.g., selection, socialization and norms, social capital, social contagion) in fostering authenticity, continuous self-improvement, and sustained performance” (Roberts, 2006, p. 293), these results open a new interesting research avenue by showing that fostering a positive climate of conflict management (i.e., open discussion of disagreements and effective resolution of conflict) can improve job performance (see also Chen et al., 2005; Greer et al., 2008; Leon-Perez et al., 2016). However, our results did not support that the existing conflict management climate in the team can enhance the positive association between task intragroup conflict and the quality of service they provide. Indeed, there was no association between task conflict and quality of service. Thus, future research should examine the role of team processes (e.g., collaboration, competition or openness: DeChurch et al., 2013; team cohesion and identity: Tekleab et al., 2009) in determining the positive or negative effects of task conflict for individual well-being and team performance. Moreover, following the positive resource caravans and gain spirals at work proposed in the Conservation of Resources (COR) theory, which assumes that various resources are salient factors in gaining new resources and enhancing well-being (Hobfoll, 1989, 2001, 2002), future research should test whether a constructive conflict management climate in the team can be considered as a team resource that not only prevents potential negative consequences of intragroup conflict on performance, but also improves relevant team processes that have potential positive consequences on team performance and viability.

Despite the above mentioned theoretical and practical implications, our study also presents some limitations. First, besides being a large and multilevel sample, we employed a cross-sectional research design; therefore future research should reply our results employing a longitudinal or cross-lagged research design. Second, regarding the measurement of conflict management climate (although we have several validity pieces of evidence; for example the bivariate correlations with the three types of conflict) we just employed a single item measure, which can explain the weak (or non-existing) moderation effect of conflict management climate. Future studies should use validated scales for measuring both conflict resolution and openness to discuss disagreements at team level. Third, like most survey-based study, our performance (quality of service) measure is perceptual in its nature and may be affected by social desirability. Further research may benefit from including objective performance indicators that combined with those perceptual will offer a more robust view of our findings.

In sum, although these results need caution since our study is not exempt from limitations, this study offers some insights into the interplay of individual resources and group dynamics, suggesting that interventions aimed both at developing individual psychological strengths and at improving group dynamics such as conflict resolution can have a positive impact on employees' well-being and performance, which are considered key indicators of positive and health organizations.

## Author contributions

JMLP participated in the study design and wrote the first draft. MA conducted the data analysis and wrote the results section. JMLR designed the study and collected the data. All wrote and revised the manuscript and agreed on the final version submitted to the journal.

## Funding

This research was funded through a research contract (Ref. 1768/0281). Authors would like to thank Mind Garden and Dr. Fred Luthans for their permission to use the PsyCap questionnaire, which is copyrighted material (see www.mindgarden.com).

### Conflict of interest statement

The authors declare that the research was conducted in the absence of any commercial or financial relationships that could be construed as a potential conflict of interest.
